# Genetic Diversities and Differentially Selected Regions Between Shandong Indigenous Pig Breeds and Western Pig Breeds

**DOI:** 10.3389/fgene.2019.01351

**Published:** 2020-01-22

**Authors:** Ming Qin, Chuanhao Li, Zhixin Li, Wei Chen, Yongqing Zeng

**Affiliations:** Shandong Provincial Key Laboratory of Animal Biotechnology and Disease Control and Prevention, College of Animal Science and Technology, Shandong Agricultural University, Tai' an City, China

**Keywords:** pigs, SLAF-seq, genetic distance, genetic variation, selection regions

## Abstract

Shandong indigenous pig breeds are an invaluable source of data on genetics in Chinese pigs. However, information on the genetic basis of these breeds remains limited. In this study, we used specific-locus amplified fragment sequencing to conduct whole-genome screening to investigate genetic diversity in Shandong indigenous breeds and Western pig breeds. The results showed that Duroc pigs (DD) had clear genetic relationships with Dapulian pigs (DPL; Fst = 0.4386) and Laiwu pigs (LW; Fst = 0.5134), and DPL and LW were relatively close genetically (Fst = 0.2334). In general, Shandong indigenous breeds showed greater genetic variety than the Western breeds. Both neighbor-joining trees and principal components analyses were able to differentiate the breeds, but population structure analyses indicated that the Western breeds genetically influenced the Shandong indigenous breeds to some extent. A total of 162 differentially selected regions (DSRs) with 841 genes and 157 DSRs with 707 genes were identified in DPL and LW, respectively. Gene annotation of the selected regions identified a series of genes regulating immunity and fat deposition. Our data confirm the rationality and accuracy of the current classification of pig breeds in Shandong province. Our results point to candidate genes in Shandong indigenous pig breeds and further promote the importance of follow-up research on functional verification.

## Introduction

Pigs are a very important domesticated species in China. The extreme climate and geographical conditions in China have contributed to the development of 88 indigenous species, many of which have unique characteristics (Yang, 2013). With an area of more than 15,000 square kilometers and a diverse terrain, Shandong province is a main center for pig production in China. Many pig breeds have been developed to meet the needs of the local people. Native breeds are characterized by strong breeding and lactation ability, good meat quality, strong adaptability, and strong resistance to disease. However, to cater to the growth performance and lean meat content associated with live pig production, modern commercial varieties were introduced in the early 20th century and crossed with local pigs (Yang et al., 2003a). Indigenous breeds from Shandong province, as well as other Chinese indigenous pig breeds, have been in direct competition with select Western breeds, primarily Duroc (DD), Yorkshire (YY), and Landrace (LL), and many of them are considered to be rare species in danger of becoming extinct. Existing indigenous pig breeds in Shandong are Heigai (HG), Dapulian (DPL), Wulian Black (WL), Laiwu (LW), Yantai Laizhou Black (YTL), Yantai Wendeng Black (YTW), Licha Black (LC), and Yimeng black pig breed (YM).

Assessing the genetic variation and population structure in Chinese indigenous pig breeds is critical in animal genetics research and government germplasm conservation. After years of effort, genetic variation and phylogenetic relationships in Chinese indigenous pig breeds have been explored morphologically, cytogenetically, and biochemically (Xiao et al., 2010). With the rapid development of molecular biology technology, research on the nuclear DNA diversity of pigs has increased, including RAPD, microsatellite DNA markers, AFLP, DNA fingerprinting, and mitochondrial DNA sequencing (Ren et al., 2000; Chen et al., 2004; Zhao et al., 2007; Wang et al., 2010). However, the majority of genetic data are still poorly characterized, except in a few very famous breeds. Of the seven pig breeds in Shandong province, only LW, YM, and LC have been investigated by microsatellite or mtDNA markers (Zhang et al., 2003; Fang et al., 2005; Megens et al., 2008). A comprehensive assessment of their genetic diversity and relationships with Western pig breeds is therefore an important step in the development of protection and improvement programs. Large-scale genotyping plays an important role in research on genetic diversity. High-throughput sequencing technology provides new opportunities for exploring gene functioning. Given its low cost, genotyping accuracy, and labeling efficiency, our study used specific-locus amplified fragment sequencing (SLAF-seq) for genotyping. This strategy, which is used in evolutionary studies on domestication, focuses on the discovery of single nucleotide polymorphisms (SNPs). SLAF-seq is generally suitable for assessing a variety of species and populations. The purpose of this study was to use SLAF-seq to determine the phylogenetic relationships among seven pig breeds in Shandong province, China, and to what extent they were influenced by Western pig breeds (DD, YY, and LL).

## Materials and Methods

### Ethics Statement

All animal care and treatment procedures were approved by the Animal Ethics Committee of Shandong Agricultural University, China, and performed in accordance with the Committee's guidelines and regulations (Approval No.: 2004006).

### Samples Collection and Genomic DNA Extraction

Ear-punch samples of 80 individuals representing 10 pig breeds were used in the study. Eight pigs were sampled from each breed except HG (n = 7), WL (n = 7), and LC (n = 10). Samples for the seven Shandong breeds were collected from conservation farms, and those for the three Western breeds were collected from a pig breeding farm in Shandong province. The number of samples per breed was determined according to the lineage of the breed. To ensure that samples covered the entire lineage of each pig population, we collected a boar from each lineage. We extracted total genomic DNA from ear-punch samples using the standard phenol-chloroform method (Sambrook et al., 2001).

### SLAF Library Preparation for Sequencing

Analyses of genomic DNA were based on SLAF-seq (Sun et al., 2013). The pig genome was selected as the reference genome for electronic enzyme digestion prediction. Our study relied on reduced representation library sequencing, which can reduce the complexity of the genome. The optimal digestion scheme was determined according to four principles: the ratio of SLAFs in the repeat sequence was as low as possible, the digested SLAFs were distributed as evenly as possible across the genome, the length of the SLAFs was highly consistent with the specific experimental system, and the number of SLAFs obtained matched the expected number of tags. These requirements elevated the efficiency of digestion. A combination of *Rsa*I and *Hae*III restriction enzymes was ultimately used (Thermo Fisher Scientific, Waltham, MA, USA) to digest pig genomic DNA into 1,010,652 SLAF tags 314 to 364 bp in length. Then, a single nucleotide (A) overhang was added to the 3′ region of the SLAF tags. To ligate dual-index sequencing adapters to the A-tailed tags, we performed restriction-ligation reactions with T4 DNA ligase (NEB). Then polymerase chain reaction (PCR) amplification was performed that included the diluted restriction-ligation DNA samples of each pig breed, dNTP, Taq DNA polymerase (NEB), and primers containing barcode 1. The PCR products were purified and run out on a 2% agarose gel. Fragments 314 to 364 bp (with indexes and adaptors) were separated. Barcode two was then added to the isolated fragments by PCR amplification. These products were purified in gel and then diluted for pair-end sequencing using an Illumina HiSeq 2500 system (Illumina, San Diego, CA, USA) according to the manufacturer's instructions.

### Read Mapping, SNP Calling, and Filtering

SLAF-seq data were processed by computer. We mapped all raw SLAF pair-end reads to the pig reference genome (Sscrofa 11.1) using BWA (Li and Durbin, 2009). In general, SLAF groups generated by reads were mapped to the same location. If the restriction enzymes only partially digested an accession, a number of reads mapped to the reference genome may have overlapped with two SLAF tags. As mentioned previously, these reads were allotted to both of the SLAF tags in the same accession. We performed SNP calling using both GATK and samtools analysis (Li et al., 2009; McKenna et al., 2010). If a locus was called by both packages, we defined it as an SNP. PLINK v1.0774 was used to filter high-quality SNPs for subsequent analysis. Minor allele frequency (MAF) evaluation was used to define alleles in each SLAF. Briefly, SNPs with integrity lower than 0.5 and MAF lower than 0.05 were excluded. A total of 314,243 highly consistent population SNPs representing 10 breeds were obtained for analyses of genetic differentiation. To avoid false positives and assess the accuracy of the digestion experiments objectively, we used rice genomic data as a control to evaluate the sequence error rate.

### Population Differentiation and Genetic Evolution Basics Analysis

The divergence index, F-statistics (Fst), is a measure of population differentiation based on genetic polymorphism data (Hudson et al., 1992). We used the PopGen package in BioPerl to evaluate Fst based on 100 kb sliding windows in 10 kb steps (Wright, 1949). We calculated the average pairwise divergence within a population (π) using a HIERFSTAT package for R. Neighbor-joining trees were constructed using MEGA 5 based on Kimura 2-parameter (Tamura et al., 2011). The bootstrap analysis (n = 1000 replications) was performed with a DISPAN package (Ota, 1993) and then beautified *via* the ITOL website (http://itol.embl.De/). The population structure was constructed with admixture v1.22 (Hubisz et al., 2009). We hypothesized that the cluster number (K value) would be between 1 and 10. We performed PCA using the smart program of EIGENSOFT (Price et al., 2006), which can assist in evolutionary analysis. To visualize the results of the algorithm analysis, we further analyzed a scatterplot of the first and second principal components. The linkage disequilibrium (r^2^) between pairwise SNPs was computed with Haploview (Barrett et al., 2004). It is generally true that r^2^ between SNPs separated by large and small genetic distance represents recent and ancient effective population sizes (Ne), respectively (Sved, 1971). In addition, we inferred the gene flow between the three Western commercial breeds and the seven Chinese indigenous pig breeds in Shandong province. We plotted migration events among the populations using TreeMix, assuming nine migration events (Wright, 1949). The covariance matrix was calculated based on 314,243 unlinked sites with a window size of an SNP.

### Detection of Selective Sweep Regions

To identify the regions that were selected during domestication, we combined the three Western pig breeds (DD, LL, and YY) into a single domestic gene pool called DLY. We used a genome-wide sliding window method to scan the selected regions with the greatest differences in genetic diversity (π log-ratio DLY/DPL and DLY/LW) and extreme divergence in allele frequency between Western commercial and Chinese indigenous pig breeds in Shandong province. To evaluate nucleotide diversity (π) and Fst, we calculated π and Fst using 100 kb windows with 10 kb steps among genomes. The top 5% π and Fst values (**Supplementary Figure S1**) between DLY and DPL and between DLY and LW in each 100 kb sliding window with a 10 kb step were used to determine potential selective-sweep regions from significantly differentiated regions.

To examine whether the candidate selective scanning regions had excessive singleton polymorphisms, we calculated Tajima's D for each pig breed using the sliding window method mentioned previously. Genes in these selective regions were identified through the Sscrofa 11.1 assembly (ftp://ftp.ensembl.org/pub/release-91/fasta/sus_scrofa/dna/) and NCBI database. In addition, we used Panther bioinformatics (www.Pantherdb.Org) for the gene function enrichment analysis.

## Results

### Specific-Locus Amplified Fragment Sequencing and SNP Discovery

**Figure 1:**
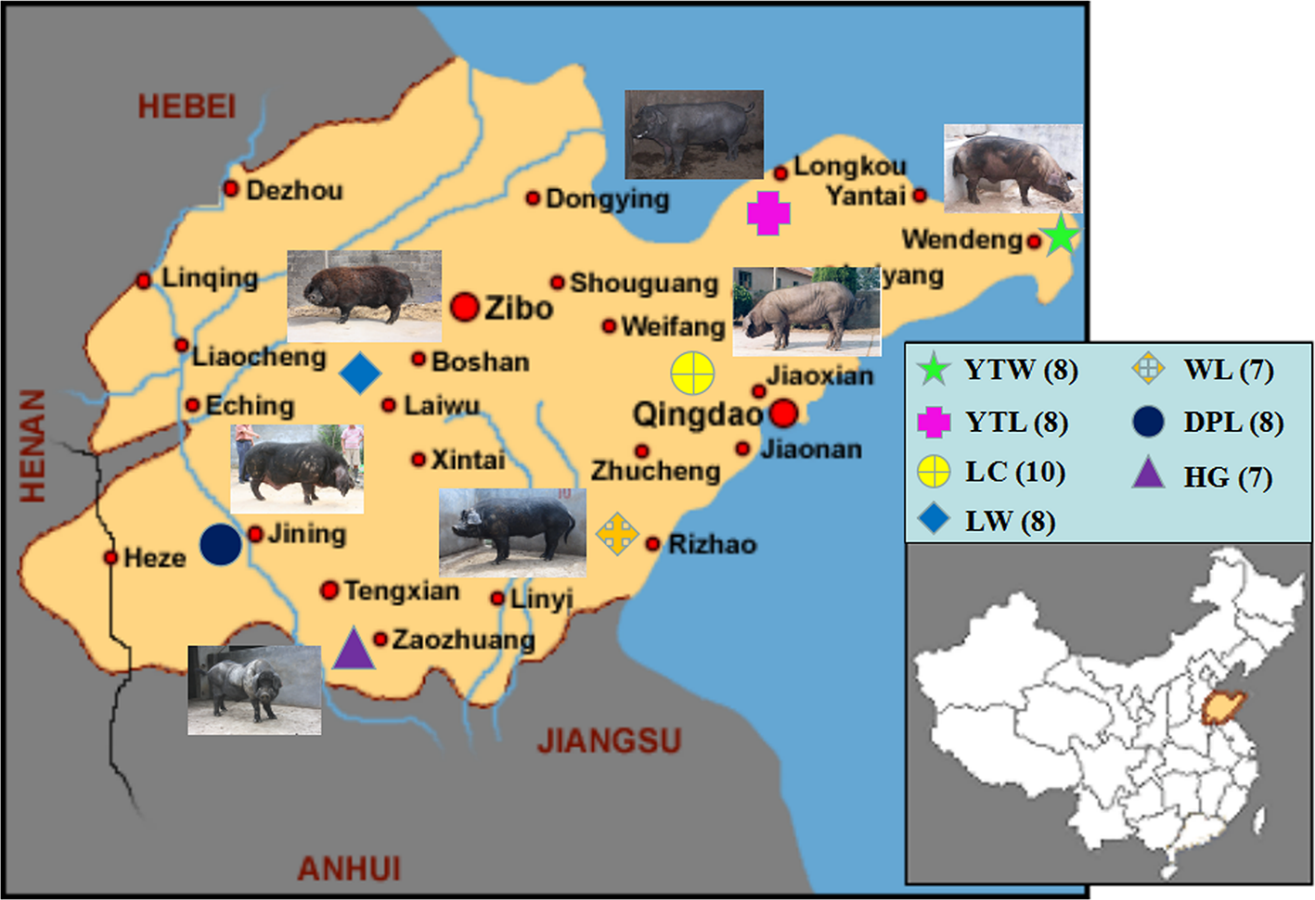
The geographical distribution of 7 Shandong indigenous pig breeds. Breeds: WL, Wulian Black pig; DPL, Dapulian pig; LW, Laiwu pig; HG, Heigai pig; YTL, Yantai Laizhou Black pig; YTW, Yantai Wendeng Black pig; LC, Licha Black pig. The following is the same.

Three of the 80 genotype samples (two HG, one YTW) were removed because the sample call rate did not meet criteria (< 95%). Nest quality control was performed on the remaining 77 samples. Of the 77 samples analyzed, 53 were from Shandong province, and 24 represented pig breeds originating outside of China (**Figure 1**).

The Sscrofa genome served as the reference genome for predicting electron enzymes and identified a restriction fragment length of Rsal + EcoRV-HF from 314 to 364 bp, which was ultimately defined as a SLAF tag. To acquire the actual SLAF marker analyzed in our study, we performed SLAF-seq in seven WL, eight DPL, eight LW, five HG, eight YTL, seven YTW, 10 LC, eight YY, eight LL, and eight DD using the same enzyme combination as in the silico restriction experiment. As shown in **Table 1**, a total of 441.19 million reads were gained from all individuals, and average Q30 and GC content was 96.26% and 39.45%, respectively. The higher Q30 value represented a lower base error rate, which means that the results of the tested sequences were reliable. Similar to the number of expected SLAFs, the total number of SLAF tags was 1,010,652. The average numbers of SLAFs and sequencing depth were 311,266.29 (13.25×), 293,802.00 (15.82×), 268,606.88 (16.82×), 251,058.20 (13.99×), 292,233.75 (16.59×), 297,446.71 (17.89×), 299,237.70 (17.49×), 271,012.13 (17.58×), 293,646.25 (18.49×), and 291,879.88 (18.70×) in WL, DPL, LW, HG, YTL, YTW, LC, YY, LL, and DD, respectively (**Table 1** and **Supplementary Table S1**). In addition, we mapped the SLAF tags to the reference genome using BWA (Li and Durbin, 2009). Moreover, when *Oryza sativa indica* was used as a control for evaluating the sequencing data, the efficiency of paired-end comparison and digestion of control were 96.97% and 95.54%, respectively, which indicates that the process was normal and available.

**Table 1:** Characteristics of SLAF-seq between Shandong indigenous pig breeds and three Western pig breeds.

Item	WL	DPL	LW	HG	YTL	YTW	LC	YY	LL	DD
Statistics of reads										
Total Reads	2990944-5966079	4019083-6070323	2975990-6381053	2650499-4682311	3867880-6470358	4454048-7384759	4208772-7544960	4391496-5628749	3713911-6903758	4557758-7187571
Average of Reads	4329274.14	4839942.63	4712400.25	3919117	5023752.88	5514933.71	5388685.20	4890713.86	5600453	5596310
GC percentage (%)	37.84-40.55	38.10-40.00	37.99-39.11	37.84-42.99	38.57-39.68	38.52-39.41	38.45-39.71	37.76-39.64	38.58-42.41	38.52-39.59
Q30 percentage (%)	94.87-95.66	94.91-95.65	95.04-95.68	95.17-95.45	95.05-95.74	94.81-95.82	94.54-95.20	94.49-95.21	94.39-94.87	94.68-95.26
Statistics of SLAFs										
SLAF number	235261-350679	273735-316650	218020-292516	191031-292759	266302-311520	273951-327892	275906-322120	239010-289822	252186-324777	265283-322884
Average of SLAFs	311266.29	293802	268606.88	251058.20	292233.75	297446.71	299233.70	271012.13	293646.25	291879.88
Average depth	13.25	15.82	16.82	13.99	16.59	17.89	17.49	17.58	18.49	18.70
Statistics of SNPs										
SNP number	797081-1160118	950380-1087829	749375-1008772	656017-1026786	923071-1053079	946837-1122484	943594-1073169	826568-977905	863697-1076420	904408-1053875
Average of SNPs	1050908.57	1013398.63	928014.88	870682.4	1002405.5	1024125.14	1007319.2	925445.25	986074.5	980454.5
Integrity (%)	48.03	46.31	42.41	39.79	45.81	46.80	46.03	42.29	45.06	44.81
Heter ratio (%)	10.52	9.83	7.86	9.03	9.20	10.28	9.81	6.92	7.27	5.84

SNP markers were defined based on the greatest depth of the sequence type as a reference sequence in each SLAF tag. A total of 8,615,537 SNPs were obtained from data from 77 samples and two wild boars downloaded from the database (ERR173221: http://www.ebi.ac.uk/ena/data/view/SAMEA1557437; ERR173222: http://www.ebi.ac.uk/ena/data/view/SAMEA1557421). The average integrity of SNPs was 44.73% (range = 29.98%–53.02%). A previous study involving SNPs (MAF < 0.05) was biased in quantifying genetic connectivity in the study of population genetic evolution (Roesti et al., 2012). To reduce any sequencing errors, eliminate baseline differentiation, and evaluate accuracy, we filtered 314,243 SNPs with MAF > 0.05 from 1,010,652 SLAFs in the 77 samples. The average number of SNPs in each breed was identified, ranging from 870,682.40 in HG to 1,050,908.57 in WL (**Table 1**). In addition, the number of SNPs in each sample was determined separately (**Supplementary Table S2**). Analyses of heterozygous loci revealed that SNP heterozygosity differed markedly by population. DD, which originated in the eastern United States, had a heterozygous ratio of only 5.84%, whereas the highest heterozygous ratio (10.52%) was found in WL.

### Genetic Variation and Genetic Distance Among Populations

The observed heterozygous number (Ho), expected heterozygous number (He), Nei diversity index (Nei), polymorphism information content (PIC), and MAF were used to determine genetic diversity. As shown in **Table 2**, all five measures revealed larger values for the Shandong indigenous breeds than the Western breeds, except for LC. Of the seven indigenous breeds in Shandong, HG had the highest genetic diversity (Ho = 0.2926, He = 0.3752, Nei = 0.3814, PIC = 0.2988, and MAF = 0.2852), whereas LW had the lowest genetic diversity (Ho = 0.2443, He = 0.37453, Nei = 0.33743, PIC = 0.2771, and MAF = 0.2585). In contrast, genetic diversity did not vary significantly among the Western pig breeds. As can be seen from **Table 2**, the average Ho ranged from 0.2249 in YY to 0.2403 in DD, and the average He ranged from 0.3259 in LL to 0.3378 in DD. It is interesting that all five calculations were higher than in the other two introduced breeds. In general, these results indicate that diversity was greater in the Shandong indigenous pig breeds than the Western breeds. Pairwise comparisons of Fst among five populations described by Weir and Cockerham are shown in **Supplementary Table S3** (Weir and Cockerham, 1984); obvious interpopulation genetic variation appeared between DD and DPL (0.4393), between DD and LW (0.5144), and between DPL and LW (0.2337), which indicates that DPL and LW have a closer genetic relationship with each other than with DD. The same was found among the YY, LL, DPL, and LW populations.

**Table 2:** The genetic variation of 7 Shandong indigenous and 3 Western pig breeds.

Breed	Ho	He	Nei	PIC	MAF
WL	0.2596	0.3497	0.3814	0.2894	0.2597
DPL	0.2711	0.3449	0.3721	0.2767	0.2579
LW	0.2443	0.3453	0.3743	0.2771	0.2585
HG	0.2926	0.3752	0.4281	0.2988	0.2852
YTL	0.2502	0.3447	0.3723	0.2771	0.2553
YTW	0.2602	0.3429	0.3735	0.2761	0.2535
LC	0.2634	0.3348	0.3548	0.2696	0.2476
YY	0.2249	0.3356	0.3625	0.2706	0.2476
LL	0.2283	0.3259	0.3511	0.2639	0.2378
DD	0.2403	0.3378	0.3639	0.2719	0.2504

Ho stands for observed heterozygous number; He, expected heterozygous number; Nei, nei diversity index; PIC, polymorphysm information content; MAF, minor allele frequency. The following is the same.

### Population Genetic Structure and Gene Flow

**Figure 2:**
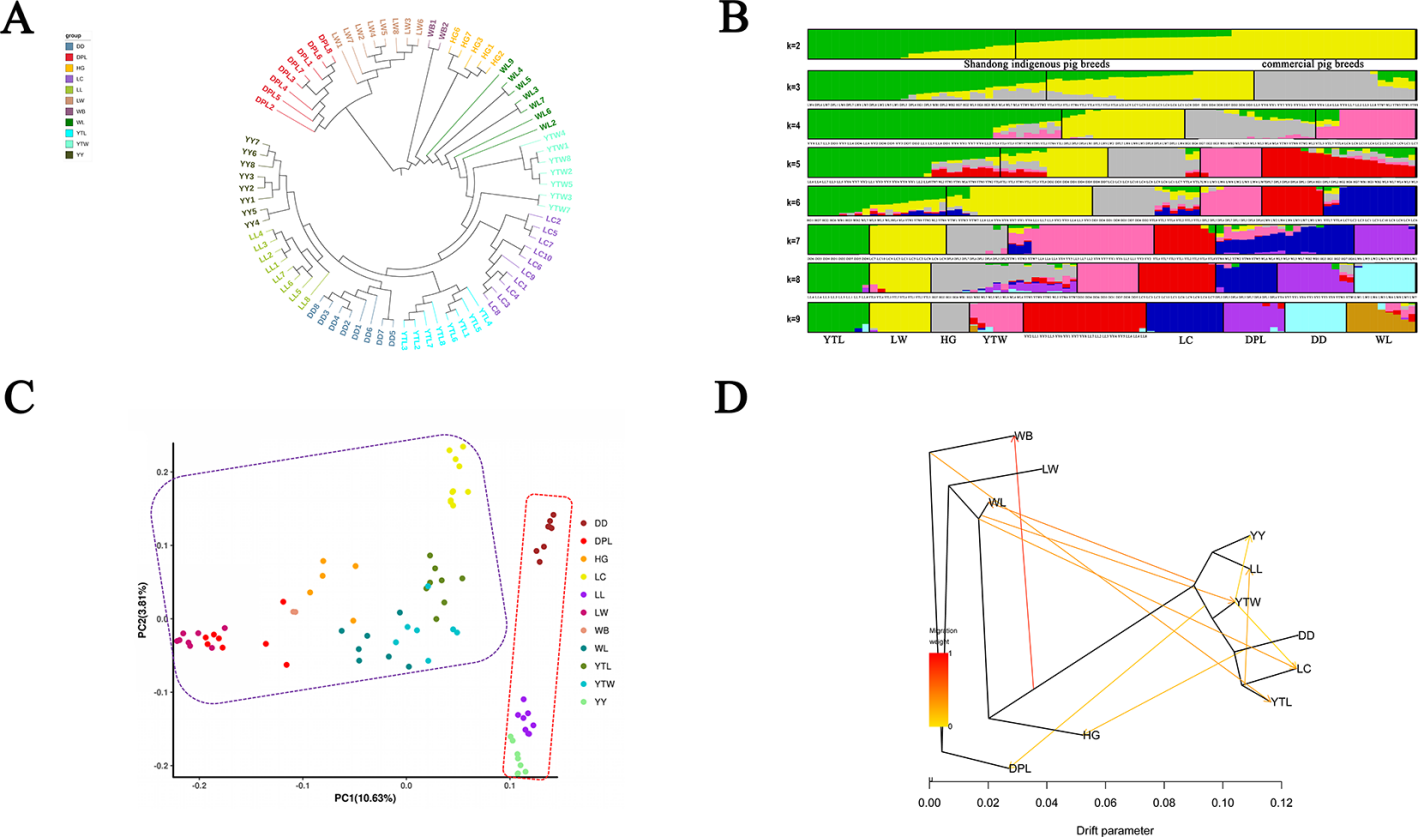
**(A)** Phylogenetic tree of Shandong indigenous pig breeds and three commercial pig breeds on our data and publicly available whole-genome sequences of pigs. **(B)** Population structure analysis of Shandong indigenous pig breeds and three commercial pig breeds on our data and publicly available whole-genome sequences of pigs. Each breed is shown as a vertical line partitioned into *K* colored components that represent inferred membership in *K* genetic clusters. **(C)** Principal component of analysis of Shandong indigenous pig breeds and three commercial pig breeds on our data and publicly available whole-genome sequences of pigs. Each dot represents sampling, and different colors represent different pig breeds. **(D)** Inferred pig tree of mixture events deduced by TreeMix. Migration arrows are colored according to their weight. Horizontal branch lengths are proportional to the amount of genetic drift that has occurred on each branch. WB, northern Chinese wild boars for which genome sequences are publicly available.

To explore genome-wide relationships and the divergence between the Shandong indigenous breeds and Western breeds, we constructed neighbor-joining trees based on pairwise genetic distance (**Figure 2A**). The trees showed that individuals of the same breed generally clustered together, which signified that they possessed unique breed identities. The results did not distinctly differentiate the Shandong and Western breeds into various clusters. Two European breeds (YY and LL) first formed a distinct outgroup, then joined with DD, and finally joined with YTL. It is noteworthy that LW and DPL were grouped together. We utilized a genetic admixture analysis to search the population structure with increasing numbers of inferred population groups (from k = 2 to k = 10). We determined based on the minimum CV error that the optimal number was K = 2 (**Supplementary Figure S2**), which was also the number of origins collected in this study. In panels with K = 2 inferred clusters, Shandong indigenous breeds and Western breeds were differentiated. When K = 9, six local breeds in Shandong (LW, HG, YTL, YTW, LC, and DPL) formed a distinct cluster, whereas the remaining breeds appeared in clusters mentioned previously in different proportions (**Figure 2B**). As expected, PCA and neighbor-joining tree analyses clearly differentiated the Shandong indigenous breeds and Western breeds (**Figure 2C**), and no distinct clusters were discovered that sustained the efficaciousness of the phylogenetic tree. Many of the geographically close breeds resembled one another. To determine the genetic contribution among the different breeds, we performed migration modeling using TreeMix. As **Figure 2D** shows, we found relatively strong gene flow between WB and a variety of indigenous and introduced breeds and relatively weak gene flow between YTW and YY, between DD and HG, and between YTL and LL.

### Accessing of Linkage Disequilibrium by r^2^

**Figure 3:**
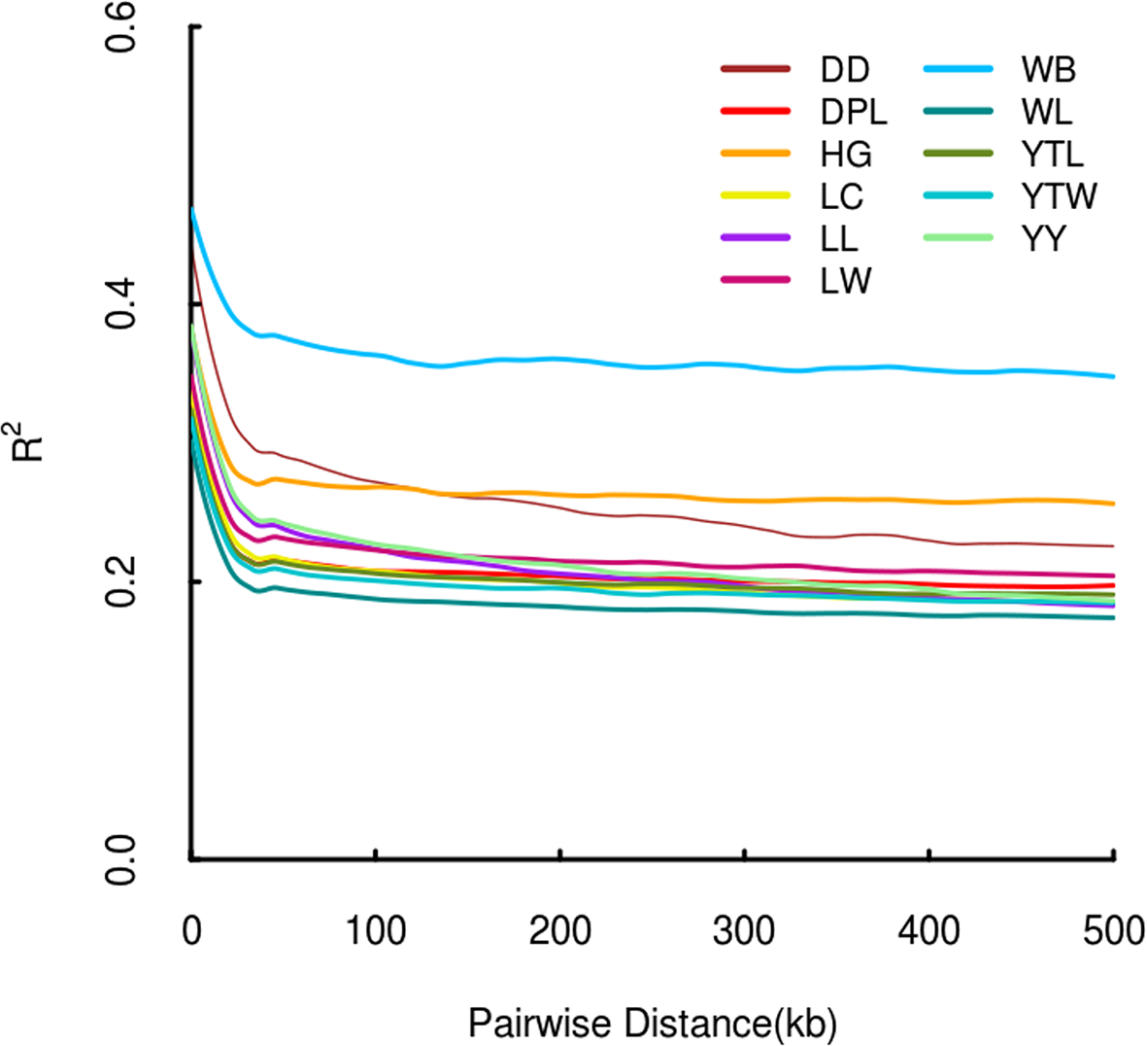
Linkage disequilibrium patterns of Shandong indigenous pig breeds and three commercial pig breeds.

The r^2^ for pairs of loci was counted for each pig breed. As physical distance increased, a similar downward trend in average r^2^ was noted in each population (**Figure 3**). As expected, LD patterns revealed that the distance of LD decay was higher in DD than DPL and LW. This shows that DPL and LW are probably subject to higher selection pressure than DD.

### Screening for Selective Sweeps in the Two Indigenous Breeds

**Figure 4:**
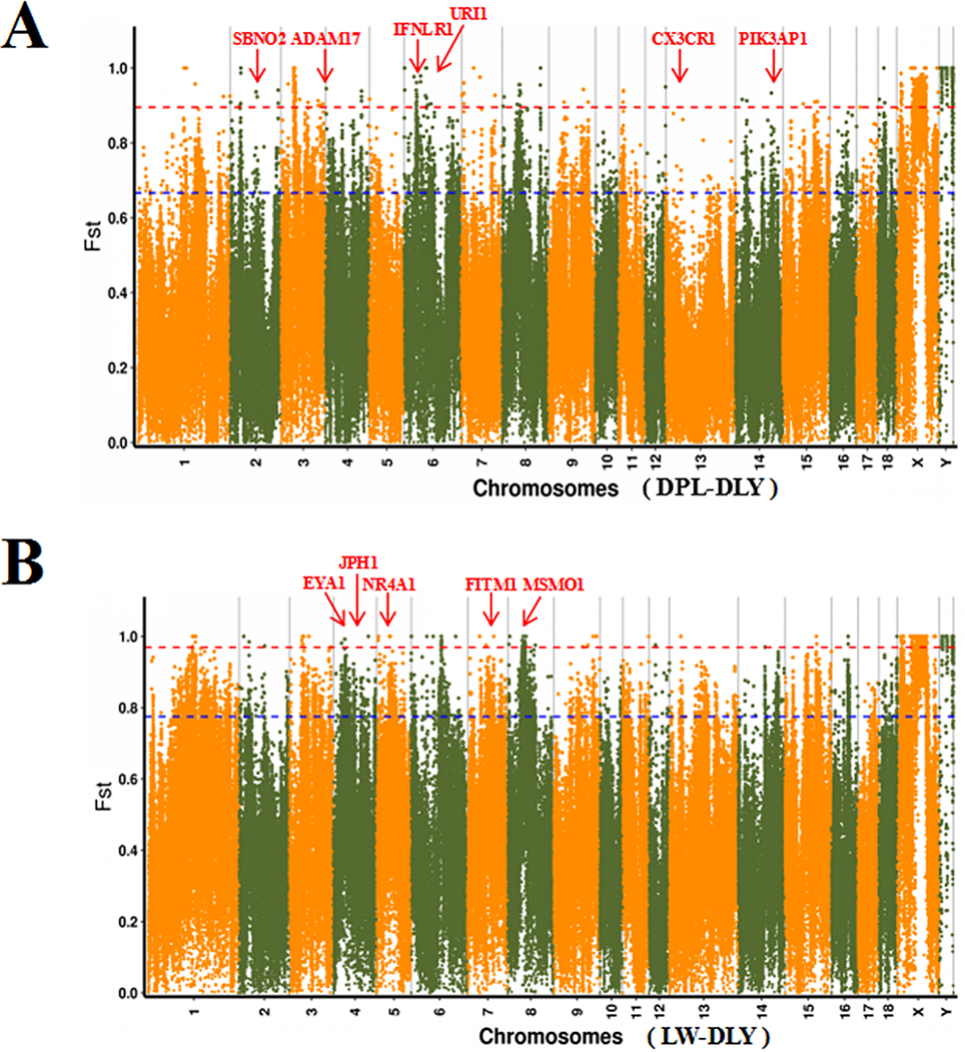
Global distribution of Fst between DPL, LW, and three Western pig breeds on autosomes. DPL-DLY represents DPL-three Western pig breeds, while LW-DLY means DPL-three Western pig breeds.

**Figure 5:**
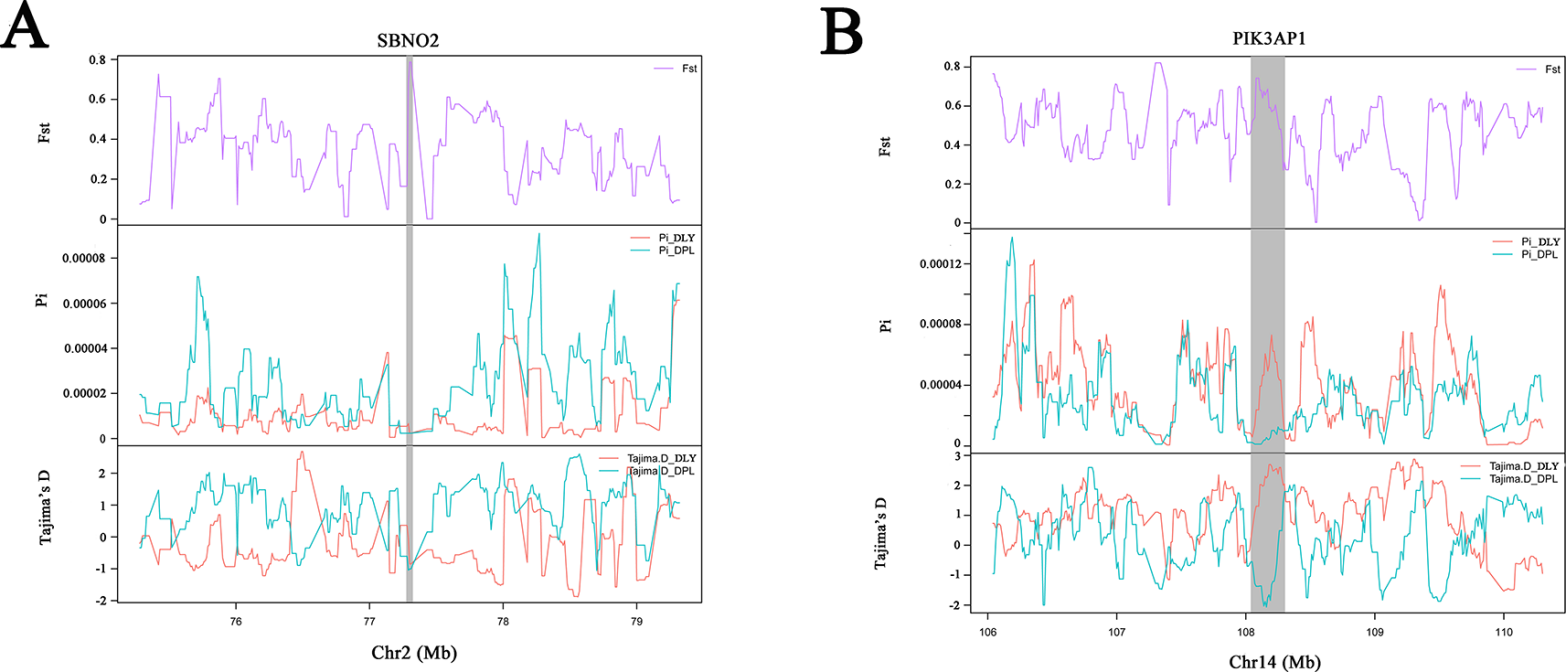
Example of genes **(A**, **B)** with selection sweep signals in DPL. Fst, Pi, and Tajima's D values are plotted. DLY (green) and DPL (blue) are represented by different colors.

To confirm the genomic regions involved in domestication, genetic diversity (π) and SNP-based Fst estimation were calculated to determine positively selected regions, defined as regions with shared differential selection signals among populations. We combined the three Western pig breeds (YY, LL, and DD) into a single gene pool and compared them to DPL and LW. A total of 162 differentially selected regions (DSRs) were shared between DPL and the commercial breeds. A further 841 unique known genes were found that contained a large number of genes related to immunity, such as *SBNO2, PIK3AP1*, and so on (**Figures 4A** and **5A**). Gene functional analyses indicated that biological processes in DPL and the three Western pig populations were enriched in T cell homeostasis, the production of tumor necrosis factor superfamily cytokines, the toll-like receptor 7 signaling pathway, and the inflammatory response (**Table 3**).

**Table 3:** The autosomal differentially selected regions (DSRs) among DPL, LW, and three Western commercial pig breeds. The partial candidate genes were given within the SNPs of the top 5% for each region.

DSRs between DPL and three Western pig breeds				
**Chr**	**Position**	**Candidate**	**GO accession**	**Description**
2	77240001-77340000	SBNO2	GO:0050727	regulation of inflammatory response
3	40350001-40490000	TMEM204	GO:0030947	regulation of vascular endothelial growth factor receptor signaling pathway
3	41450001-41630000	POLR3K	GO:0051607	defense response to virus
3	59060001-59160000	GNLY	GO:0050832	defense response to fungus
3	76690001-76790000	ACTR2	GO:0038096	Fc-gamma receptor signaling pathway involved in phagocytosis
3	126840001-126950000	ADAM17	GO:0032722	positive regulation of chemokine production
4	83570001-83710000	CD247	GO:0050852	T cell receptor signaling pathway
5	21260001-21380000	RAB5B	GO:0030100	regulation of endocytosis
6	29430001-29590000	GNAO1	GO:0007212	dopamine receptor signaling pathway
6	37750001-38030000	MYLK3	GO:0002528	regulation of vascular permeability involved in acute inflammatory response
6	40110001-40240000	URI1	GO:0009615	response to virus
6	51600001-51800000	EXOC3L2	GO:0006887	exocytosis
6	53320001-53470000	EHD2	GO:0006897	endocytosis
6	72100001-72240000	TNFRSF8	GO:0042108	positive regulation of cytokine biosynthetic process
6	79380001-79480000	ECE1	GO:0001921	positive regulation of receptor recycling
6	81890001-81990000	IFNLR1	GO:0050691	regulation of defense response to virus by host
9	33220001-33340000	MMP7	GO:0050830	defense response to Gram-positive bacterium
13	23860001-23970000	CX3CR1	GO:0070098	chemokine-mediated signaling pathway
14	31430001-31660000	P2RX7	GO:0006954	inflammatory response
14	108030001-108220000	PIK3AP1	GO:0050727	regulation of inflammatory response
18	24840001-24960000	CADPS2	GO:0045921	positive regulation of exocytosis
**DSRs between LW and three Western pig breeds**				
**Chr**	**Position**	**Candidate**	**GO accession**	**Description**
3	80750001-80850000	PEX13	GO:0001561	fatty acid alpha-oxidation
4	61560001-61660000	JPH1	GO:0007517	muscle organ development
4	63870001-63980000	EYA1	GO:0014706	striated muscle tissue development
5	17230001-17410000	NR4A1	GO:0035914	skeletal muscle cell differentiation
7	75050001-75180000	FITM1	GO:0034389	lipid particle organization
8	43640001-43740000	MSMO1	GO:0006633	fatty acid biosynthetic process
11	68810001-68940000	ZIC5	GO:0030154	cell differentiation
13	108990001-109100000	SKIL	GO:0007519	skeletal muscle tissue development
13	197680001-197790000	SLC5A3	GO:0015798	myo-inositol transport
14	121360001-121490000	ADRA2A	GO:0050995	negative regulation of lipid catabolic process
14	131210001-131320000	FGFR2	GO:0051150	regulation of smooth muscle cell differentiation

Genome-wide scans of LW and the three commercial breeds are shown in **Figure 4B**. A total of 157 DSRs were discovered in these groups. A further 707 unique known genes were discovered, such as *NR4A1, MSMO1*, and so on (**Figure 5B**). Gene enrichment analyses revealed that DSRs in LW and the three foreign breeds were involved in the regulation of smooth muscle cell differentiation, muscle organ development, lipid particle organization, and negative regulation of lipid catabolic processes (**Table 3**).

## Discussion

In this research, we used SLAF-seq to explore population structure and genetic diversity in Shandong local pig breeds and modern commercial pig breeds. SLAF-seq uses reduced representation library sequencing for *de novo* SNP and precise genotyping (Li and Durbin, 2009). Compared to traditional mark development methods, SLAF-seq has several significant advantages: there is no need to reference the genomic sequence and polymorphic information; it is highly efficient; it is inexpensive; and it saves time. In addition, research methods, such as mitochondrial genome sequencing, focus on a limited range of molecular markers of the genome. SLAF-seq, in contrast, has better resolution and provides more genetic information based on high-density and uniform coverage of the entire gene. Genomic data have provided new insights into plant domestication (Jiang et al., 2015; Han et al., 2016; Yang et al., 2018). In recent years, genomic data have been used to screen positive selection signals in dogs and chickens (Axelsson et al., 2013; Wang et al., 2016). However, to date, there has been little research on genetic relationships between local pig breeds in Shandong province and outside breeds involving large-scale analyses of genomic data.

In this study, we investigated seven pig breeds collected from all growing regions of Shandong province, ensuring adequate representativeness of the sample. With an average sequencing depth of 16.93-fold for all individuals, 8,615,537 SNPs were identified from 10 pig populations by SLAF-seq. Previous studies have shown that the accuracy of evolutionary analysis can be guaranteed when the sequencing depth reaches 5-fold (Li et al., 2013). This result is several times the number of SNPs obtained from Illumina PorcineSNP60 BeadChip genotyping. To more fully understand genetic diversity and population structure in Taihu indigenous pigs, researchers identified a total of 105,550 SNPs with MAF ≧ 0.05 using GGRS (Wang et al., 2015). By comparison, 314,243 filtered SNPs were available for analysis in our study. Therefore, SLAF-seq can produce more information on genomic variation.

The YY, LL, and DD pig breeds originated in the United Kingdom, Denmark, and the United States, respectively. This indicates that the geographical distance of indigenous pig breeds in Shandong province is generally greater than that of Western breeds. As anticipated, both population structure and cluster analyses demonstrated that the Western breeds were genetically distant from the Shandong indigenous breeds (**Figure 2B**). Furthermore, pairwise Fst estimation showed that DD was more genetically distant than DPL and LW (Fst = 0.4386 for DPL, Fst = 0.5134 for LW), which indicates tremendous genetic differentiation between DD and other local pig breeds. Our sequencing results are consistent with data from other genetic markers, such as mitochondrial DNA and microsatellite markers (Yang et al., 2003a; Yang et al., 2003b), which have also revealed that European and Chinese indigenous pig breeds are genetically distant. LW and DPL were grouped in a separate cluster in the neighbor-joining trees (**Figure 2A**) with a close genetic distance of 0.2337, which is consistent with the results of previous mitochondrial DNA sequencing (Wang et al., 2009; Wang et al., 2010). Firstly, the main distribution areas of Laiwu and Dapulian are contiguous. Although the establishment of conservation farms has been relatively perfect, there is still a small cross between them. Secondly, there is frequent gene flow among them. As we know that in some conservation farms of them, phenotypically alike indigenous pigs of other breeds are sometimes introduced to reduce the inbreeding of their population. These results show that Shandong indigenous pig breeds have great genetic variety. We speculate that the Western pig breeds are subject to strong human selection pressure. It also may be due to the fact that there were more indigenous pigs than commercial pigs. To summarize, this study supplies a theoretical basis for elucidating patterns in genetic diversity and population structure in local pig breeds and Western pig breeds.

When we examined the migration patterns of different pig breeds using TreeMix (Pickrell and Pritchard, 2012), we discovered different degrees of genetic contribution between indigenous breeds in Shandong and Western breeds. The analyses showed obvious gene flow from YTW to YY and from YTL to LL. Yantai is located in the northeastern Shandong Peninsula, where the economy and culture are relatively advanced and Western pig breeds have long been introduced. In contrast, Laiwu and Dapulian have been relatively less influenced by Western pig breeds. The two indigenous breeds are distributed mainly in the middle of Shandong province, where traffic and economic development are lagging. Si et al. recorded that LW were influenced by YY in 1950, but beyond that, DPL was hybridized with Middle Yorkshire in the 1950s and 1960s. In other words, LW and DPL are more protected and less influenced by the Western pig breeds.

Shandong indigenous pig breeds are a good model for identifying meaningful signatures of selection on genes reflecting economic traits in pig at the genome level. DPL is distributed mainly in Jining, Shandong province, China. Historically, DPL was well known for properties related to resistance and immunity. This study used the thresholds of Fst and π to identify DSRs. Fst is more convincing for identifying complicated events, such as detection of significant variation (Innan and Kim, 2008). A large number of key genes potentially related to immunity, such as *SBNO2, IFNLR1, CX3CR1, ADAM17, URI1*, and *PIK3AP1*, were found in the DSR data sets. Previous results indicated that *SBNO2* participates in anti-inflammatory response by inhibiting the NF-kB pathway (El Kasmi et al., 2007). However, little is known about the function of *SBNO2* in porcine resistance to disease. Our results demonstrate that *SBNO2* may play an important role in porcine resistance to inflammation, but this hypothesis requires further investigation and validation. *IFNLR* belongs to the type II cytokine receptor family. It can bind with type III interferons (IFN-λs) to participate in signal transduction and induce antiviral and anti-tumor functions (Wang et al., 2019). Recently, more and more research has indicated that *IFNLR1* is also widely expressed in immune cells. In viral infection or acute inflammation, IFN-λ can directly or indirectly act on immune cells to play a role in immunoregulation (Ank et al., 2008; Lazear et al., 2015). Therefore, our data might provide insight into the role of candidate genes in porcine immunity.

LW is representative of a typical Shandong black pig. As it has fresh meat and a high proportion of intramuscular fat (Chen et al., 2017), it serves as a perfect model for tracking selection for fat deposition in muscle. We therefore investigated DSRs under selection between LW and the Western pig breeds. Gene annotation of the selected regions revealed a series of functional genes for fat deposition and muscle development. It is interesting to note that we discovered some candidate genes (i.e., *EYA1, NR4A1, MSMO1, FITM1*, and *JPH1*). *EYA1*, a homolog of Drosophila eyes absent, is expressed in the mesenchyme (Xu et al., 1999). *EYA1* may play a key role in the genetic hierarchy of kidney organogenesis (Sajithlal et al., 2005). Moreover, the synergistic effect of *SIX1* and *EYA1* can promote transformation of slow-twitch to fast-twitch phenotype in the muscles of adult mice (Grifone et al., 2004). As skeletal muscle fibers affect the tenderness of pork, *EYA1* may be a special gene regulating the quality of pork. Our data also indicate that *NR4A1* might contribute to regulating fat metabolism. It has been reported that *NR4A1* affects glucose and lipid metabolism in muscle (Maxwell et al., 2005; Chao et al., 2007). At present, most studies indicate that *NR4A1* has an inhibitory effect on fatty buildup. However, the specific and complete regulatory mechanisms remain to be verified. In a word, our data provide clues for identifying genetic differences between LW and Western pig breeds.

LC, YTL, and YTW have a very close genetic relationship. At the genome level, the genetic difference between LC and Western pig breeds is still largely unknown. Results of KEGG pathway analyses have shown that most genes are involved in the chemokine signaling pathway, the Ras signaling pathway, and tyrosine metabolism. Some key genes (i.e., *SLC9A1* and *TIAM1*) in the regulation of actin cytoskeleton, and a novel gene designated 2-acylalycerol O-acyltransferase 2-A in fat digestion and absorption, were discovered in pathway analyses. Other studies have shown that the DH domain of *TIAM1* plays an important role in cell deformation and cytoskeletal reorganization (Habets et al., 1994; Michiels et al., 1997). Our results might provide new insights into their function in affecting pig growth and development. To date there is still a lack of conservation of WL, which continues to be crossbred with Western pig breeds. Nonetheless, our findings indicate that many positive selection genes are involved in the MAPK signaling pathway in WL, such as *MAP3K12, RPS6KA1, RAPGEF2*, and *MKNK2*. MAPK is very important for mediating inflammation and cytokine production. In addition, through sequencing, we verified that HG has its own unique genetic characteristics and is an independent indigenous pig population. Similarly, we found that *KDR* is involved in the PI3K-AKT pathway in HG. Our data suggest that *MAP3K12* and *KDR* might regulate the porcine inflammatory response, but additional studies are needed to confirm this.

In sum, the genetic structure and relationships between Shandong indigenous pig breeds and three Western breeds were revealed by SLAF-seq. Our results provide additional information explaining differences in economic characteristics between the breeds. Our research therefore not only provides a cost-effective strategy for pig genome-wide screening but also lays a genetic foundation for further research on the candidate gene functions of DSRs.

## Data Availability Statement

The raw data supporting the conclusions of this article will be made available by the authors, without undue reservation, to any qualified researcher.

## Ethics Statement

All animal care and treatment procedures were approved by the Animal Ethics Committee of Shandong Agricultural University, China. All methods were carried out in accordance with the guidelines and regulations of the Animal Ethics Committee of Shandong Agricultural University (Approval number: 2004006).

## Author Contributions

WC and YZ conceived and designed the research. WC, MQ, CL, and ZL performed the research. MQ, WC, and CL analyzed the data. YZ contributed reagents and materials. MQ wrote the manuscript. WC and YZ provided substantial comments and revised the manuscript. All authors read and approved the final version of the manuscript.

## Funding

This study was supported financially by the National Natural Science Foundation of China (No. 31401055), the Funds of Shandong “Double Tops” Program (No. SYL2017YSTD12), the Shandong Provincial Modern Pig Technology and Industry System Project (No. SDAIT-08-02), Shandong Provincial Natural Science Foundation (No. ZR2018BC046, ZR2019MC053).

## Conflict of Interest

The authors declare that the research was conducted in the absence of any commercial or financial relationships that could be construed as a potential conflict of interest.
