# Supplementary material for: Genetic Diversities and Differentially Selected Regions Between Shandong Indigenous Pig Breeds and Western Pig Breeds
Source: Front Genet. 2020 Jan 22;10:1351. doi: 10.3389/fgene.2019.01351 (PMC6987402; doi:10.3389/fgene.2019.01351)
Supplement: Supplementary file 1 [file DataSheet_1.doc]

***Supplementary Material***

**Supplementary Figures and Tables**


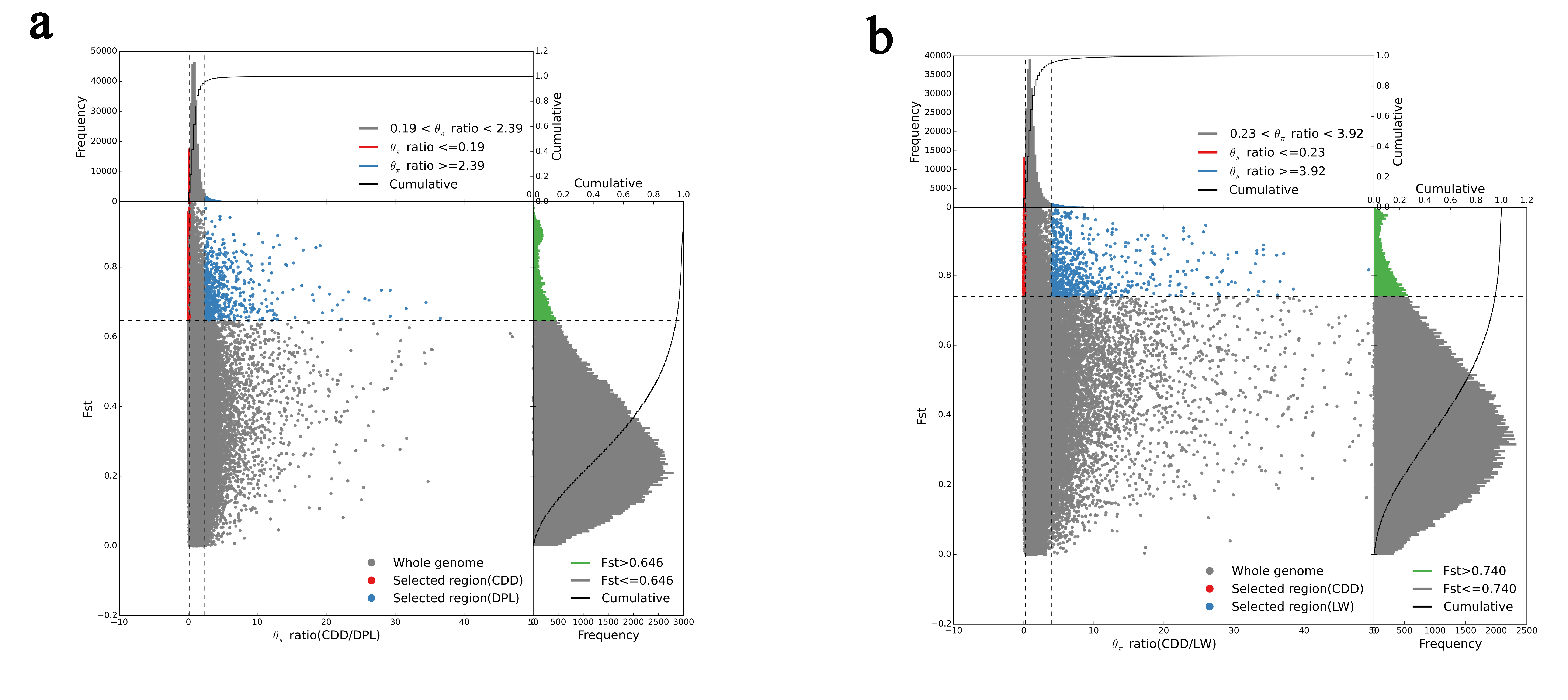


**Supplementary Figure 1.** The top 5% for π and Fst value between DLY and DPL (A), between DLY and LW B).

Note: Red and blue are selected sites that are screened by π greater than 95% quantiles and less than 5% quantiles, and Fst greater than 95% quantiles.


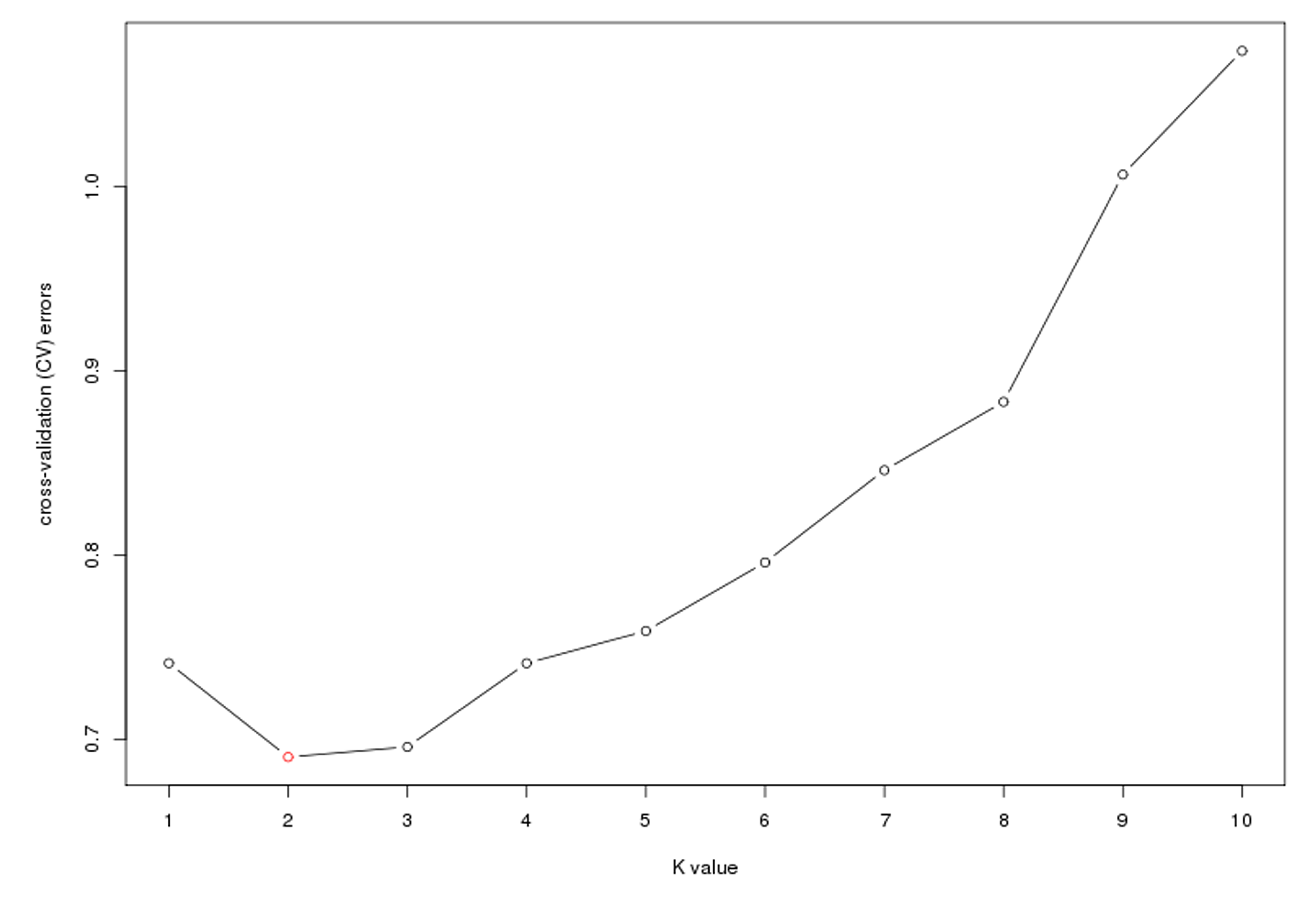


**Supplementary Figure 2.** Cross‐validation error rates corresponding to different *K* values show *K* = 2 is the best.

**Supplementary Table 1.** Characteristics of SLAF tags between Shandong indigenous pig breeds and Western pig breeds

| Sample ID | BMK ID | SLAF number | Total depth | Average depth |
| --- | --- | --- | --- | --- |
| WLH2 | aa | 341435 | 3997615 | 11.7083 |
| WLH3 | ab | 338242 | 4463924 | 13.1974 |
| WLH4 | ac | 350679 | 5730460 | 16.341 |
| WLH5 | ad | 293426 | 3480334 | 11.861 |
| WLH6 | ae | 346264 | 4500239 | 12.9966 |
| WLH7 | af | 273557 | 3915867 | 14.3146 |
| DPL1 | ag | 293557 | 4384419 | 14.9355 |
| DPL2 | ah | 313600 | 5847511 | 18.6464 |
| DPL3 | ai | 316650 | 4286846 | 13.5381 |
| DPL4 | aj | 290589 | 4527475 | 15.5803 |
| DPL5 | ak | 283105 | 4472863 | 15.7993 |
| DPL6 | al | 295369 | 5038717 | 17.0591 |
| DPL7 | am | 273735 | 3840504 | 14.03 |
| DPL8 | an | 283811 | 4802792 | 16.9225 |
| LW1 | ao | 292516 | 6171903 | 21.0994 |
| LW2 | ap | 279106 | 3994925 | 14.3133 |
| LW3 | aq | 269848 | 4401125 | 16.3096 |
| LW4 | ar | 273074 | 4588683 | 16.8038 |
| LW6 | as | 274728 | 5256027 | 19.1317 |
| LW7 | at | 259399 | 4844207 | 18.6747 |
| HG2 | au | 280066 | 4430008 | 15.8177 |
| HG3 | av | 292054 | 3370583 | 11.541 |
| HG7 | ax | 292759 | 4518427 | 15.4339 |
| YTL1 | ay | 294704 | 4331651 | 14.6983 |
| YTL2 | az | 306641 | 4197389 | 13.6883 |
| YTL3 | ba | 266302 | 3724789 | 13.9871 |
| YTL4 | bb | 301407 | 4757600 | 15.7846 |
| YTL5 | bc | 272143 | 4407465 | 16.1954 |
| YTL6 | bd | 278700 | 5209727 | 18.693 |
| YTL8 | be | 311520 | 5946260 | 19.0879 |
| YTW1 | bf | 290538 | 5666750 | 19.5043 |
| YTW2 | bg | 295361 | 4756248 | 16.1032 |
| YTW3 | bh | 289859 | 4629780 | 15.9725 |
| YTW4 | bi | 290584 | 4309428 | 14.8302 |
| YTW5 | bj | 327892 | 7169981 | 21.8669 |
| YTW7 | bl | 313942 | 6615029 | 21.0709 |
| YTW8 | bm | 273951 | 4348468 | 15.8732 |
| LCH1 | bn | 289039 | 4100189 | 14.1856 |
| LCH2 | bo | 302824 | 5018926 | 16.5737 |
| LCH3 | bp | 299541 | 4119359 | 13.7522 |
| LCH4 | bq | 290490 | 4770365 | 16.4218 |
| LCH5 | br | 317784 | 7360031 | 23.1605 |
| LCH6 | bs | 304316 | 4893539 | 16.0805 |
| LCH7 | bt | 322120 | 5658869 | 17.5676 |
| LCH8 | bu | 310647 | 7078924 | 22.7877 |
| LCH9 | bv | 279670 | 4527069 | 16.1872 |
| LCH10 | bw | 275906 | 5017104 | 18.1841 |
| DB1 | bx | 283822 | 5429771 | 19.1309 |
| DB2 | by | 289822 | 4696867 | 16.206 |
| DB3 | bz | 280041 | 4920565 | 17.5709 |
| DB4 | ca | 239010 | 4286082 | 17.9326 |
| DB5 | cb | 264132 | 4394232 | 16.6365 |
| DB6 | cc | 261374 | 4272720 | 16.3472 |
| DB7 | cd | 270056 | 4622061 | 17.1152 |
| DB8 | ce | 279840 | 5510014 | 19.6899 |
| CB1 | cf | 306311 | 5731231 | 18.7105 |
| CB2 | cg | 309658 | 6736957 | 21.7561 |
| CB3 | ch | 292580 | 5235986 | 17.8959 |
| CB4 | ci | 324777 | 6347255 | 19.5434 |
| CB5 | cj | 282412 | 4809801 | 17.0311 |
| CB6 | ck | 252186 | 3635193 | 14.4147 |
| CB7 | cl | 288739 | 5205208 | 18.0274 |
| CB8 | cm | 292507 | 6004965 | 20.5293 |
| DLK1 | cn | 288418 | 5817207 | 20.1694 |
| DLK2 | co | 290125 | 5490097 | 18.9232 |
| DLK3 | cp | 303107 | 5727857 | 18.8971 |
| DLK4 | cq | 283028 | 4448763 | 15.7185 |
| DLK6 | cr | 278711 | 5114231 | 18.3496 |
| DLK7 | cs | 265283 | 4872614 | 18.3676 |
| DLK8 | ct | 303483 | 5292358 | 17.4387 |
| WLH9 | da | 235261 | 2908737 | 12.3639 |
| LW5 | db | 282164 | 4233481 | 15.0036 |
| LW8 | dc | 218020 | 2889269 | 13.2523 |
| HG1 | dd | 199381 | 2710403 | 13.5941 |
| HG6 | df | 191031 | 2592431 | 13.5707 |
| YTL7 | dg | 306453 | 6316566 | 20.6119 |
| DLK5 | dh | 322884 | 7014492 | 21.7245 |
| total 1010652 |  |  |  |  |

**Supplementary Table 2.** Characteristics of SNPs between Shandong indigenous pig breeds and Western pig breeds

| #Sample | SNPnumber | Deficiency | HeterLociNum | HomoLociNum | Hetloci-ratio | Integrity-ratio |
| --- | --- | --- | --- | --- | --- | --- |
| R01 | 240335 | 73908 | 36276 | 204059 | 15.09% | 76.48% |
| R02 | 263813 | 50430 | 49924 | 213889 | 18.92% | 83.95% |
| aa | 274806 | 39437 | 58114 | 216692 | 21.14% | 87.45% |
| ab | 278993 | 35250 | 61851 | 217142 | 22.16% | 88.78% |
| ac | 280457 | 33786 | 63319 | 217138 | 22.57% | 89.24% |
| ad | 256453 | 57790 | 50423 | 206030 | 19.66% | 81.60% |
| ae | 277694 | 36549 | 61240 | 216454 | 22.05% | 88.36% |
| af | 254008 | 60235 | 52240 | 201768 | 20.56% | 80.83% |
| ag | 263057 | 51186 | 55050 | 208007 | 20.92% | 83.71% |
| ah | 270654 | 43589 | 60297 | 210357 | 22.27% | 86.12% |
| ai | 274355 | 39888 | 49429 | 224926 | 18.01% | 87.30% |
| aj | 259822 | 54421 | 54499 | 205323 | 20.97% | 82.68% |
| ak | 261022 | 53221 | 49173 | 211849 | 18.83% | 83.06% |
| al | 263716 | 50527 | 48613 | 215103 | 18.43% | 83.92% |
| am | 245437 | 68806 | 44310 | 201127 | 18.05% | 78.10% |
| an | 256542 | 57701 | 43141 | 213401 | 16.81% | 81.63% |
| ao | 261568 | 52675 | 47032 | 214536 | 17.98% | 83.23% |
| ap | 248456 | 65787 | 41897 | 206559 | 16.86% | 79.06% |
| aq | 247161 | 67082 | 35414 | 211747 | 14.32% | 78.65% |
| ar | 248138 | 66105 | 36813 | 211325 | 14.83% | 78.96% |
| as | 249112 | 65131 | 38282 | 210830 | 15.36% | 79.27% |
| at | 238378 | 75865 | 40455 | 197923 | 16.97% | 75.85% |
| au | 253331 | 60912 | 46594 | 206737 | 18.39% | 80.61% |
| av | 252917 | 61326 | 51236 | 201681 | 20.25% | 80.48% |
| ax | 257790 | 56453 | 47891 | 209899 | 18.57% | 82.03% |
| ay | 261517 | 52726 | 47829 | 213688 | 18.28% | 83.22% |
| az | 264100 | 50143 | 50380 | 213720 | 19.07% | 84.04% |
| ba | 239869 | 74374 | 38001 | 201868 | 15.84% | 76.33% |
| bb | 265009 | 49234 | 50132 | 214877 | 18.91% | 84.33% |
| bc | 246453 | 67790 | 43285 | 203168 | 17.56% | 78.42% |
| bd | 248926 | 65317 | 46849 | 202077 | 18.82% | 79.21% |
| be | 272211 | 42032 | 42512 | 229699 | 15.61% | 86.62% |
| bf | 266998 | 47245 | 48402 | 218596 | 18.12% | 84.96% |
| bg | 268424 | 45819 | 55529 | 212895 | 20.68% | 85.41% |
| bh | 268083 | 46160 | 51954 | 216129 | 19.37% | 85.31% |
| bi | 264361 | 49882 | 49243 | 215118 | 18.62% | 84.12% |
| bj | 284639 | 29604 | 61173 | 223466 | 21.49% | 90.57% |
| bl | 285534 | 28709 | 61556 | 223978 | 21.55% | 90.86% |
| bm | 264927 | 49316 | 50727 | 214200 | 19.14% | 84.30% |
| bn | 264334 | 49909 | 42863 | 221471 | 16.21% | 84.11% |
| bo | 277679 | 36564 | 51568 | 226111 | 18.57% | 88.36% |
| bp | 276984 | 37259 | 50985 | 225999 | 18.40% | 88.14% |
| bq | 271916 | 42327 | 49340 | 222576 | 18.14% | 86.53% |
| br | 284833 | 29410 | 56114 | 228719 | 19.70% | 90.64% |
| bs | 277193 | 37050 | 48277 | 228916 | 17.41% | 88.20% |
| bt | 284823 | 29420 | 51366 | 233457 | 18.03% | 90.63% |
| bu | 281626 | 32617 | 45022 | 236604 | 15.98% | 89.62% |
| bv | 266864 | 47379 | 49249 | 217615 | 18.45% | 84.92% |
| bw | 264221 | 50022 | 43584 | 220637 | 16.49% | 84.08% |
| bx | 270761 | 43482 | 36253 | 234508 | 13.38% | 86.16% |
| by | 260942 | 53301 | 31925 | 229017 | 12.23% | 83.03% |
| bz | 264857 | 49386 | 32709 | 232148 | 12.34% | 84.28% |
| ca | 235524 | 78719 | 26518 | 209006 | 11.25% | 74.94% |
| cb | 255672 | 58571 | 33006 | 222666 | 12.90% | 81.36% |
| cc | 251578 | 62665 | 29373 | 222205 | 11.67% | 80.05% |
| cd | 260023 | 54220 | 32364 | 227659 | 12.44% | 82.74% |
| ce | 258755 | 55488 | 37286 | 221469 | 14.40% | 82.34% |
| cf | 274797 | 39446 | 34447 | 240350 | 12.53% | 87.44% |
| cg | 275487 | 38756 | 39311 | 236176 | 14.26% | 87.66% |
| ch | 266383 | 47860 | 36911 | 229472 | 13.85% | 84.76% |
| ci | 277940 | 36303 | 44262 | 233678 | 15.92% | 88.44% |
| cj | 264414 | 49829 | 36086 | 228328 | 13.64% | 84.14% |
| ck | 243285 | 70958 | 27141 | 216144 | 11.15% | 77.41% |
| cl | 265456 | 48787 | 34276 | 231180 | 12.91% | 84.47% |
| cm | 268230 | 46013 | 34831 | 233399 | 12.98% | 85.35% |
| cn | 267995 | 46248 | 29417 | 238578 | 10.97% | 85.28% |
| co | 268607 | 45636 | 28870 | 239737 | 10.74% | 85.47% |
| cp | 274796 | 39447 | 28002 | 246794 | 10.19% | 87.44% |
| cq | 262763 | 51480 | 26542 | 236221 | 10.10% | 83.61% |
| cr | 262969 | 51274 | 27374 | 235595 | 10.40% | 83.68% |
| cs | 252678 | 61565 | 27496 | 225182 | 10.88% | 80.40% |
| ct | 275057 | 39186 | 31982 | 243075 | 11.62% | 87.53% |
| da | 209862 | 104381 | 31003 | 178859 | 14.77% | 66.78% |
| db | 236845 | 77398 | 30200 | 206645 | 12.75% | 75.37% |
| dc | 197406 | 116837 | 24023 | 173383 | 12.16% | 62.81% |
| dd | 184614 | 129629 | 22459 | 162155 | 12.16% | 58.74% |
| df | 177152 | 137091 | 21811 | 155341 | 12.31% | 56.37% |
| dg | 265676 | 48567 | 49209 | 216467 | 18.52% | 84.54% |
| dh | 263258 | 50985 | 28473 | 234785 | 10.81% | 83.77% |
| Total | 314243 |  |  |  |  |  |

**Supplementary Table 3.** The genetic distance among two Chinese indigenous breeds and three Western pig breeds

|  | DPL | LW | HG | WL | LC | YTW | YTL | YY | LL | DD |
| --- | --- | --- | --- | --- | --- | --- | --- | --- | --- | --- |
| DPL |  |  |  |  |  |  |  |  |  |  |
| LW | 0.2344 |  |  |  |  |  |  |  |  |  |
| HG | 0.2535 | 0.3041 |  |  |  |  |  |  |  |  |
| WL | 0.2179 | 0.2754 | 0.1451 |  |  |  |  |  |  |  |
| LC | 0.2876 | 0.3548 | 0.2184 | 0.1637 |  |  |  |  |  |  |
| YTW | 0.2403 | 0.3021 | 0.1541 | 0.0831 | 0.1358 |  |  |  |  |  |
| YTL | 0.2702 | 0.3376 | 0.1767 | 0.1333 | 0.1500 | 0.1116 |  |  |  |  |
| YY | 0.3938 | 0.4611 | 0.2946 | 0.2054 | 0.2443 | 0.1707 | 0.2053 |  |  |  |
| LL | 0.3906 | 0.4601 | 0.2876 | 0.2053 | 0.2246 | 0.1797 | 0.1920 | 0.1587 |  |  |
| DD | 0.4386 | 0.5134 | 0.3243 | 0.2671 | 0.2559 | 0.2191 | 0.2160 | 0.2719 | 0.2543 |  |
